# A New Pepstatin-Insensitive Thermopsin-Like Protease Overproduced in Peptide-Rich Cultures of *Sulfolobus solfataricus*

**DOI:** 10.3390/ijms15023204

**Published:** 2014-02-21

**Authors:** Marta Gogliettino, Alessia Riccio, Ennio Cocca, Mosè Rossi, Gianna Palmieri, Marco Balestrieri

**Affiliations:** Institute of Biosciences and BioResources, National Research Council (CNR-IBBR), Via Pietro Castellino 111, Naples 80131, Italy; E-Mails: marta.gogliettino@ibbr.cnr.it (M.G.); alessia.riccio@ibbr.cnr.it (A.R.); ennio.cocca@ibbr.cnr.it (E.C.); m.rossi@ibp.cnr.it (M.R.); marco.balestrieri@ibbr.cnr.it (M.B.)

**Keywords:** *Sulfolobus solfataricus*, thermopsin, thermophilic enzyme, signal-transduction pathways

## Abstract

In this study, we gain insight into the extracellular proteolytic system of *Sulfolobus solfataricus* grown on proteinaceous substrates, providing further evidence that acidic proteases were specifically produced in response to peptide-rich media. The main proteolytic component was the previously isolated SsMTP (*Sulfolobus solfataricus* multi-domain thermopsin-like protease), while the less abundant (named SsMTP-1) one was purified, characterized and identified as the *sso1175* gene-product. The protein revealed a multi-domain organization shared with the cognate SsMTP with a catalytic domain followed by several tandemly-repeated motifs. Moreover, both enzymes were found spread across the *Crenarchaeota* phylum and belonging to the thermopsin family, although segregated into diverse phylogenetic clusters. SsMTP-1 showed a 75-kDa molecular mass and was stable in the temperature range 50–90 °C, with optimal activity at 70 °C and pH 2.0. Serine, metallo and aspartic protease inhibitors did not affect the enzyme activity, designating SsMTP-1 as a new member of the pepstatin-insensitive aspartic protease family. The peptide-bond-specificity of SsMTP-1 in the cleavage of the oxidized insulin B chain was uncommon amongst thermopsins, suggesting that it could play a distinct, but cooperative role in the protein degradation machinery. Interestingly, predictions of the transmembrane protein topology of SsMTP and SsMTP-1 strongly suggest a possible contribution in signal-transduction pathways.

## 1. Introduction

Most heterotrophic hyperthermophiles are able to grow on proteinaceous substrates as primary carbon and energy sources [1,2]. Although the details of metabolic schemes involved in peptide utilization by these organisms are still unknown, it can be hypothesized that a general degradation–uptake–conversion process occurs. Enzymatic pathways for the assimilating metabolism of these substrates must initially require extracellular proteases [3–7], in the form of cell membrane-associated, as well as free released enzymes.

Extracellular hydrolysis generates peptides in a wide range of molecular mass that need to be transported into the cell by specific systems; the concerted action of proteases and peptidases located inside the cell can allow for the further hydrolysis of the internalized peptides to individual amino acids [8,9]. Many Archaea, such as *Thermoproteus tenax*, *Archaeoglobus fulgidus* and *Sulfolobus solfataricus*, are supposed to oxidize peptides to CO_2_, using sulfur, thiosulfate, sulfate, oxygen, nitrite and nitrate as possible terminal electron acceptors [10,11]. The recent boost of research activity on the genome sequence of these organisms should allow for the accurate analysis and reconstruction of at least some steps of these pathways.

*S. solfataricus* is an obligate aerobe that grows in hot and acidic environments either chemolithotrophically, by oxidizing metal cations (Fe^2+^) or sulfur, as well as heterotrophically on simple sugars. It originates from a solfataric field with temperatures between 75 and 90 °C and pH values of 1.0–3.0 [12,13]. Within its environment, *Sulfolobus* can interact with a complex ecosystem consisting of a variety of primary producers and decomposers of organic matter. Although *S. solfataricus* has been reported to grow on a wide variety of reduced organic compounds as the sole carbon and energy source [13], the nutrient utilization by this microorganism requires complex mechanisms of uptake and metabolism that remain not yet well defined. The metabolic pathways for the degradation of sugars have been studied in detail [14,15], and several reports indicate that *S. solfataricus* predominantly uses ATP-binding cassette (ABC) transporter systems for the uptake of carbohydrate compounds [16,17]. In contrast, little is known about the molecular physiology of *S. solfataricus* when peptides are provided as the sources of carbon and energy.

In the present study, the patterns of extracellular free and cell surface-associated proteins, which were expressed at the early stationary phase by *S. solfataricus* grown in the presence or absence of different sources of peptides, were comprehensively analyzed; this comparative approach was aimed at elucidating the peptide-induced strategy adopted by this microorganism to support growth and cell survival in response to specific environmental stimuli. When the complex proteinaceous substrates were added to the *S. solfataricus* cultures, the total extracellular protease activity strongly increased with respect to cultures in a basal medium, suggesting that the expression of proteolytic components can be specifically induced in response to the nutrient composition of the growth media. Specifically, under these growth conditions, the *S. solfataricus* P2 strain exhibited the production of a new thermopsin-like protease, named SsMTP-1. This enzyme represents a novel type of thermostable, pepstatin-insensitive acid protease, showing optimal activity at high temperatures and extremely acidic pHs.

This study contributes to the basic knowledge of the extracellular proteases produced by *S. solfataricus* in peptide-rich media and possibly involved in cell nutrition and signaling, which allows microorganisms to sense environmental modifications and adapt to their ecological niche.

## 2. Results and Discussion

### 2.1. Cell Growth and Analysis of Extracellular Protease Activities

As previously reported, SDS-polyacrylamide gel electrophoresis (SDS-PAGE) and zymographic analyses of exoproteins in *S. solfataricus* cultures showed a protein pattern and a profile of proteases in peptide-rich media (supplemented with tryptone, yeast extract and sucrose, TYS) significantly different from those observed in yeast extract and sucrose (YS) basal media [7]. In addition, in TYS culture, an extracellular membrane-bound protease (SsMTP) over-produced in response to this peptide-rich nutrient was purified and characterized, revealing a new member of the thermopsin family [7]. Therefore, with the aim of further investigating the *S. solfataricus* extracellular proteolytic enzymes, we decided to analyze the effects of different proteinaceous sources on the protease production, as it is known that the high content of complex organic substances promotes cell growth and protease biosynthesis.

**Figure 1.**
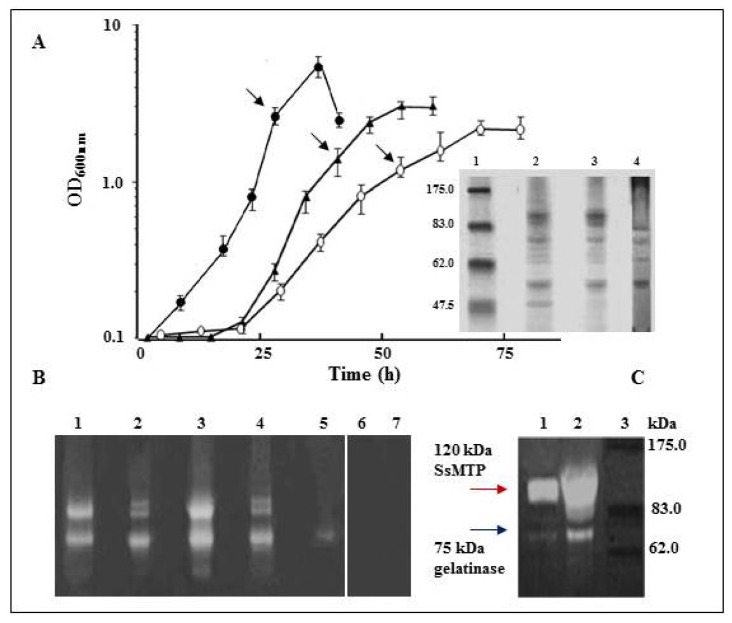
Cell growth and analysis of extracellular protease activities. (**A**) Growth kinetics of *S. solfataricus* at 80 °C in YS (yeast extract and sucrose; white circle), TYS (tryptone, yeast extract and sucrose; triangle) or PYS (peptone, yeast extract and sucrose; black circle) supplemented media. The arrows indicate the stages at which the cells were harvested for SDS-polyacrylamide gel electrophoresis (SDS-PAGE) analysis. (**insert**): Lane 1, molecular weight markers; Lane 2, extracellular proteins from PYS cultures; Lane 3, extracellular proteins from TYS cultures; and Lane 4, extracellular proteins from YS cultures; (**B**) Gelatin zymogram under non-denaturing conditions of extracellular proteins from TYS, PYS and YS cultures. Lanes 1–2, 9 U of total protease activity released into PYS and TYS media, respectively; Lanes 3–5, 7.0 μg of total extracellular protein from PYS, TYS and YS cultures, respectively; and Lanes 6–7, control samples of concentrated PYS or TYS culture broth, respectively; and (**C**) Analysis of the extracellular TYS-proteome by SDS-gelatin zymogram. Lane 1, extracellular proteins from TYS culture pre-warmed at 60 °C for 1 h; and Lane 2, extracellular proteins from TYS culture not thermally denatured; and Lane 3, molecular weight markers.

As shown in Figure 1A, the addition of peptone, yeast extract and sucrose (PYS) to the basal medium significantly increased the cell density at the stationary phase of growth with respect to TYS media, leading to a reduction of the doubling time. Moreover, the SDS-PAGE analysis (Figure 1A) of the extracellular proteins from TYS or PYS cultures at the late exponential phase revealed a similar pattern, with the overproduction of distinct protein bands, undetectable in YS basal media (Figure 1A). However, under these peptide-rich growth conditions, the extracellular protease-specific activities, detected at very acidic pHs, were 16,000 U/mg (using hemoglobin as the substrate) or 3.0 U/mg (using Z-Gly-pNPE (N-CBZ-glycine *p*-nitrophenyl ester) as the substrate) in TYS cultures and 3800 U/mg (using hemoglobin) or 0.5 U/mg (using Z-Gly-pNPE) in PYS cultures, indicating that the tryptic digest of casein was the most effective inducer of protease production. In contrast, no proteolytic activity was revealed in YS cultures using the substrates mentioned above. These findings reasonably suggest that extracellular, but not cytoplasmic, proteases contribute to the proteolytic activity measured in the culture broths.

The production of proteases in the two different media was further investigated by zymographic analyses performed under native conditions. The presence of discrete bands positive for gelatin-hydrolysis (not observed in the culture media before the addition of *S. solfataricus* cells) was shown only after gel incubation at pH 3.0 (Figure 1B), while no signals were detected in the pH range 6.0–8.0, indicating that the *S. solfataricus* extracellular proteolytic enzymes belong to the acid protease cluster. Moreover, an evident increase in the intensity of the proteolytic bands was revealed in TYS with respect to PYS media, either normalizing against total protein or activity levels (Figure 1B), possibly due to a different peptide composition of the two supplemented nutrients. Indeed, tryptone is a caseinolytic peptide mixture from trypsin digestion, while peptone is a pepsin digest of animal tissues, which is highly rich in low molecular weight peptides and amino acids.

A more accurate analysis of the extracellular TYS-proteome by SDS-gelatin zymogram (Figure 1C) showed two main activity bands, the most abundant being a 120 kDa gelatinase, corresponding to the already characterized SsMTP and a 75 kDa gelatinase, whose relative intensity increased during cell growth until the stationary phase (data not shown). These findings provide physiological and biochemical evidence that protein degradation products in *S. solfataricus* occur mainly by means of extracellular acidic proteases.

### 2.2. Purification of the 75 kDa Gelatinase from Tryptone, Yeast Extract and Sucrose (TYS) Culture

**Figure 2.**
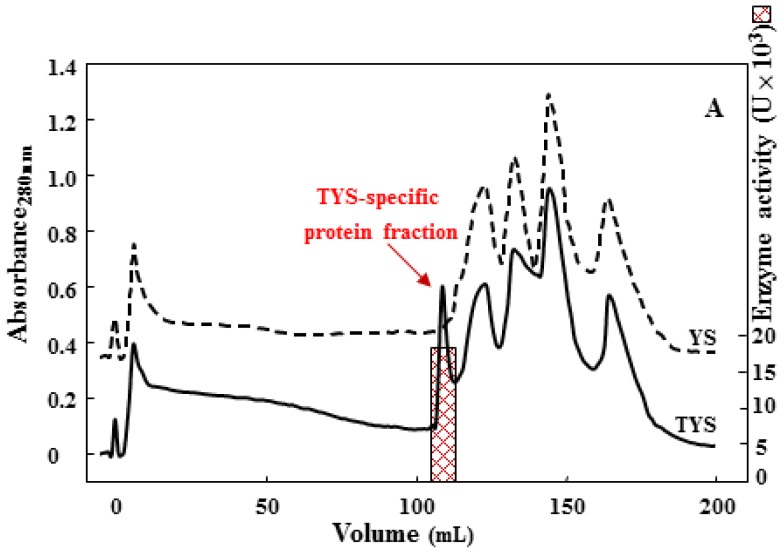

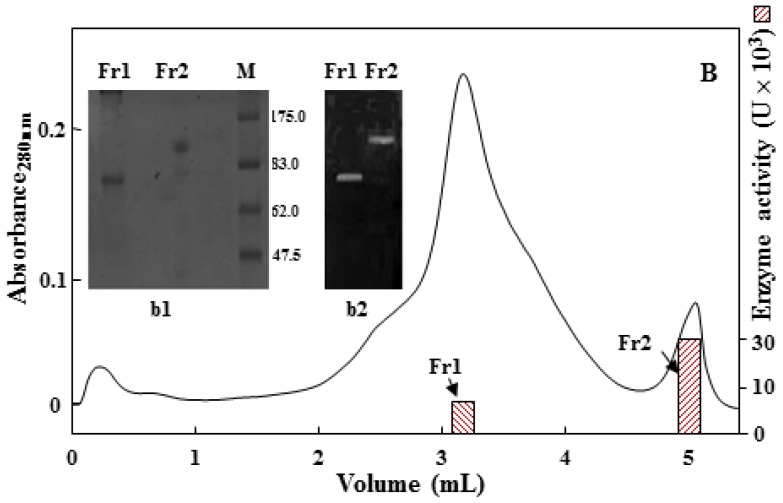
Purification of 75 kDa gelatinase from TYS culture. (**A**) Diethylaminoethyl (DEAE)-anion-exchange chromatography of extracellular protein from TYS (solid line) and YS (dot line) cultures. The bar indicates the protease activity using hemoglobin as the substrate; and (**B**) Hydrophobic affinity chromatography of the protease activity fractions eluted from DEAE chromatography. Fr1 and Fr2 protease activity fractions (dashed bars) were analyzed by SDS-PAGE (**b1 insert**) and gelatin-SDS zymogram (**b2 insert**).

With the aim of identifying the protein associated with the 75 kDa gelatinase band, a purification strategy was assessed. In the setting up of a separation procedure, the choice of the first chromatographic step was critical to obtain the effective isolation of the acidic protease. Therefore, by way of a combination of anion exchange (diethylaminoethyl, DEAE) and hydrophobic affinity (phenyl Superose) chromatography, a new protease was purified, and the homogeneity of the final product was determined by SDS-PAGE and *N*-terminal sequence analyses. Interestingly, the chromatographic profile of TYS-exoproteins on a DEAE column (Figure 2A) revealed the presence of a unique protease active fraction, which was not observed in the corresponding analysis of YS-exoproteins (Figure 2A). A further purification step on a phenyl Sepharose column (Figure 2B) allowed the separation of two distinct activity fractions eluted at 0.3 (Fr1) and 0.05 M (Fr2) (NH_4_)_2_SO_4_, showing 75 and 120 kDa molecular mass, respectively, as determined by SDS-PAGE (**b1 insert**) and SDS-gelatin zymogram analyses (**b2 insert**). A summary of the purification procedure is reported in Supplementary Table S1.

The 120 kDa protease was confirmed to be the previously characterized SsMTP, while the 75 kDa enzyme was revealed to be the protein encoded by the gene *sso1175* (GenBank Accession ID. AAK41423.1), on the basis of Edman degradation analysis, which gave the *N*-terminal sequence VYTSGITFY. This *N*-terminus matched a region starting from position +25 on the *sso1175* gene-product, thus indicating the occurrence of polypeptide maturation through signal peptide cleavage during protein export. The putative mature protein contains 576 amino acid residues with a calculated molecular mass of 65.103 Da and a theoretical pI of 6.21. Moreover, a homology search of the *sso1175-*product showed that this protein shared a significant sequence identity (30%–40%) only with some thermopsins (specifically from *Sulfolobus* species and *Acidianus hospitalis*, *Caldisphaera lagunensis* and *Thermoproteus tenax*), the typical acid proteases found in the archaeal kingdom. On the other hand, a low sequence identity (about 15%) was revealed between the purified protease and the cognate SsMTP previously described from *Sulfolobus solfataricus*. Thermopsins, recognized by their specific inhibition by pepstatin, have been identified in a variety of microorganisms and belong to the aspartic family, which shows optimum pH in the acidic range (pH 3–4). However, pepstatin-insensitive acid proteases, especially from *Xanthomonas* sp., *Pseudomonas* sp., *Bacillus* sp. and *Thermoplasma volcanium* have also been reported [18].

### 2.3. Structural and Phylogenetic Analyses

**Figure 3.**
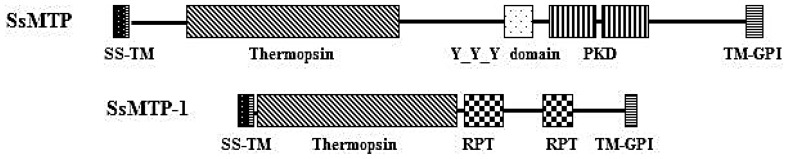
Domain architecture of the SsMTP and SsMTP-1 precursors using the SMART program. The domain structure is schematized: SS, signal sequence; TM, transmembrane domain; Thermopsin, thermopsin-like catalytic domain; PKD, polycystic kidney disease domain; RPT, Repeat Pattern Toolkit; Y_Y_Y, two component regulator three Y domain; and TM-GPI, transmembrane domain, including the glycosylphosphatidylinositol signal sequence.

An analysis of the protein architecture of SSO1175 showed a multiple domain organization, similar to that observed for SsMTP [**7**], including a putative signal sequence (predicted by the Signal P algorithm) [19], spanning the residues, M1–T20, a thermopsin-like domain (A25–R271 residues) and two internal repeat regions (Repeat Pattern Toolkit, RPT; V297–N375 and V446–N523, respectively), which are located at the *C*-terminal portion of the protein (Figure 3). A more detailed investigation revealed that the RPT domains are widely found in all kingdoms of life and supposedly play a regulatory role in protein evolution and function through protein-protein interaction processes. Most of these protein domains are located in the extracellular side, but their function remains not completely clarified. However, it is believed that the importance of repeats in understanding biological function resides not only in their high frequency among known sequences, but also in their abilities to confer multiple binding and structural roles on proteins, providing opportunities for the organism to expand its repertoire of cellular functions [20]. Concerning the *C*-terminal end, a specific sequence motif required for the glycosylphosphatidyl inositol (GPI) lipid attachment of proteins to the cytoplasmic membrane, was identified in the *sso1175*-product sequence spanning the residues A582–R601, suggesting the occurrence of GPI modification machinery, as previously proposed for archaeal proteins [21,22]. Therefore, the SSO1175 can be considered a new member of the *S. solfataricus* multi-domain thermopsin-like protease (SsMTP) family and is thus named SsMTP-1. This protease is one of the 30 archaeal components of the “predicted thermopsin-like proteins” belonging to arCOG03671 of the Clusters of the Orthologous Groups database, which is a framework for providing orthologous relationships from completely sequenced genomes. This cluster is different from that including the SsMTP and all the other conserved archaeal thermopsins (creCOG01712; 15 proteins).

**Figure 4.**
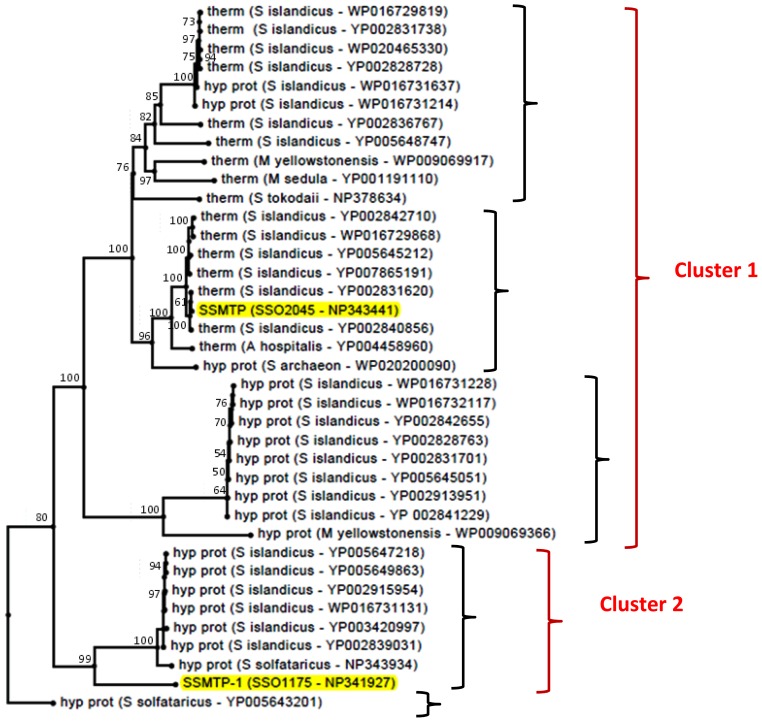
Phylogenetic analysis of SsMTP-1 and SsMTP in the archaeal kingdom (highlighted). The cladogram includes the homologous sequences retrieved from all the archaeal organisms. The numbers at nodes represent the confidence limits computed by the bootstrap procedure (100 replicates). Black curly brackets indicate the five thermopsin-like protein families defined by the phylogenetic tree. Red curly brackets indicate the two clusters, including SsMTP (Cluster 1) or SsMTP-1 (Cluster 2).

To investigate the distribution of SsMTP and SsMTP-1 homologs in the entire archaeal kingdom, a phylogenetic tree was constructed. The cladogram reported in Figure 4 was obtained by a user-friendly web service (http://www.phylogeny.fr/), dedicated to reconstructing and analyzing phylogenetic relationships between molecular sequences through the connection of several programs recognized for both speed and accuracy (see the Experimental Section). Following this approach, putative SsMTP and SsMTP-1 homologs (Supplementary Figure S1) were found to be restricted to the *Crenarchaeota* phylum and subdivided into five families, revealing the occurrence of SsMTP-1 in an evolutionary lineage different from that including the cognate SsMTP and the typical archaeal thermopsins. Moreover, all the retrieved protein homologs were found clustered into two main groups: the first one **(**creCOG01712) containing the SsMTP and all the other conserved archaeal thermopsins and the second one (arCOG03671), including SsMTP-1 and several hypothetical proteins, which are not biochemically characterized and not assigned to any known protease family. Therefore, on the basis of the sequence homology and phylogenetic analysis, we hypothesized that SsMTP and SsMTP-1 may play a different physiological role in *S. solfataricus* cell growth.

### 2.4. Enzymatic Properties of SsMTP-1 Protease

The optimal temperature for SsMTP-1 activity was found to be 70 °C, and the optimal pH was 2.0 (Supplementary Figure S2A,B), which falls within the range already established for the other extracellular acid proteases (pH 2.0–4.5). Moreover, an investigation into thermal stability revealed that the enzyme was thermostable in the temperature range 50–90 °C (Supplementary Figure S2C), similar to the cognate SsMTP [7]. In order to classify the nature of the active site, we explored the effect of different compounds on the ability of SsMTP-1 to hydrolyze hemoglobin as a substrate. As expected, neither serine-(phenylmethylsulfonyl fluoride (PMSF)) nor metallo-(ethylenediamine tetraacetic acid (EDTA)) protease inhibitors produced any effects on enzyme activity. Surprisingly pepstatin, a well-known inhibitor of aspartic proteases and thermopsins, did not affect SsMTP-1 activity, even in a stoichiometric excess, suggesting that this enzyme could be a new member of the pepstatin-insensitive acid protease group [18].

**Figure 5.**
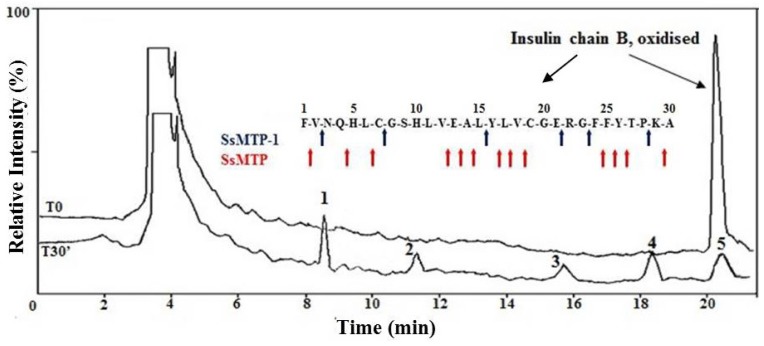
Cleavage site specificity of SsMTP-1. Overlay of the total ion current chromatograms by high-performance liquid chromatography (HPLC)-electrospray mass spectrometry analysis at time 0 (T0) and 30 min (T30) of SsMTP-1 incubated with the oxidized form of the bovine insulin B chain at 70 °C and pH 2.0. The *y*-axis represents the relative intensity in percentage measured in counts per second (cps) and normalized to the bovine insulin B chain peak height observed at T0. Cleavage site specificity of SsMTP-1, indicated by arrows on the peptide substrate, was shown in the insert. The data relative to SsMTP were taken from Cannio *et al.* [7].

To study the cleavage specificity of SsMTP-1, the bovine oxidized insulin B chain was chosen as the peptide substrate model, since in this protein, a useful variety of bonds available for cleavage are freely accessible. Direct identification of the hydrolysis products generated from the oxidized insulin B chain was performed by high-performance liquid chromatography (HPLC)-electrospray mass spectrometry (Figure 5) [23]. Each experiment was stopped after 30 min of incubation at 70 °C using a 500 substrate/enzyme (S/E; *w*/*w*) ratio. SsMTP-1 showed endopeptidase activity, as verified by the analysis of mass spectrometry (MS), which revealed the presence of the entire peptide substrate (1–30) and five distinct signals related to the peptides 8–21, 3–7, 24–28, 24–30 and 16–30 (Supplementary Table S2). Therefore, the results of this analysis allowed for the identification of the specific enzymatic cleavage sites (Figure 5): Val-Asn, Cys-Gly, Leu-Tyr, Glu-Arg, Gly-Phe and Pro-Lys. Interestingly, in contrast to the previously characterized SsMTP [7], the preferential peptide bonds hydrolyzed by SsMTP-1 appeared similar to those of pepsin, an endopeptidase with a broad specificity, which prefers large hydrophobic residues at both sides of the scissile bond. Therefore, the two SsMTP proteases display complementary and no overlapping specificities that could improve protein degradation efficiency under acidic conditions

### 2.5. Computational Protein Function Predictions

**Figure 6.**
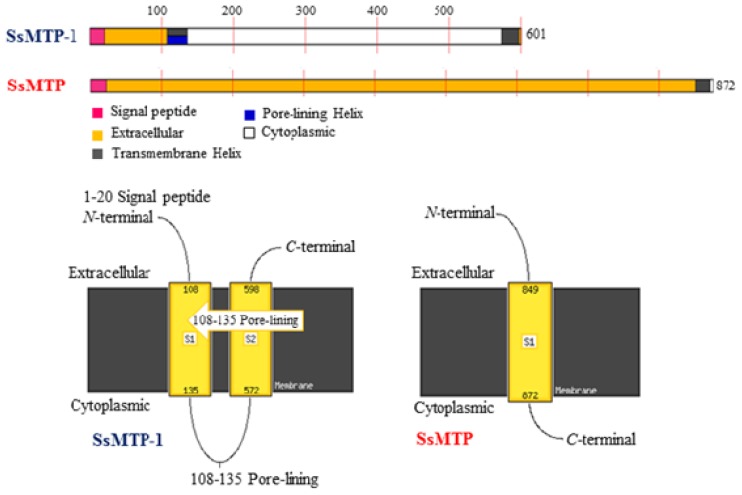
The computational protein function predictions of SsMTP-1 and SsMTP. The schematic diagram of the MEMSAT-SVM (Membrane Helix Prediction) prediction and corresponding transmembrane topology.

By using a combined neural network and model recognition approach of the PSIPRED Protein Sequence Analysis Workbench (http://bioinf.cs.ucl.ac.uk/psipred/), a protein structural prediction for functional classification was performed. This analysis showed a different membrane topological organization for SsMTP and SsMTP-1, suggesting a possible involvement of these proteases in the cell surface signaling pathways (Figure 6). However, as previously mentioned, the two proteases were found to be released into TYS conditioned media, possibly due to a kind of cell surface “proteolytic shaving” [24]. Indeed, a previous proteomic study [25] identified in the *S. solfataricus* cell surface fraction a cluster of *N*-glycosylated proteins, including SsMTP (SSO2045) and SsMTP-1 (SSO1175), as also confirmed by the SDS-gelatin zymogram analysis (data not shown).

The cellular signaling processes are poorly studied in the archaeal kingdom, and very little information on the role of proteases in these pathways has been reported so far [26]. Adaptation to changing internal and environmental circumstances requires that living organisms develop mechanisms for triggering compensatory changes in the functional status of target proteins. The process by which sensory information is received and translated into cellular effects is called signal transduction, which can be achieved via a cascade of proteolytic events and is responsible for the appropriate response of the cell to a wide range of environmental conditions. In this context, it is still unknown whether archaeal microorganisms have developed unique paradigms for transducing signals comparable to those of Bacteria or Eukarya or novel ways to exploit the known mechanisms.

In view of the results obtained, we suggest that thermopsins and, particularly, the sub-family, whose representative member is SsMTP-1, could play an important role in the response of *Crenarchaeota* to changes in environmental conditions.

## 3. Experimental Section

### 3.1. Enzymes and Reagents

α-Cyano-4-hydroxycinnamic acid was purchased from Sigma (Milano, Italy). Trifluoroacetic acid (TFA), HPLC grade, was from Carlo Erba (Milano, Italy). All other reagents and solvents of the highest purity were available from Baker (Milano, Italy).

### 3.2. Organism, Culture Conditions and Protein Production

*S. solfataricus* strain P2 (DSM 1617) was grown at 80 °C in glycine buffered Brock’s medium [12] with 0.05% yeast extract and 0.2% sucrose at pH 3.2 (YS basal medium) or in YS supplemented with 0.1% tryptone (TYS) or 0.1% peptone (PYS). The optical density of liquid cultures was monitored at 600 nm, and the culture media were harvested at the stationary phase (1.2 Optical Density, OD). For solid media, gellan gum (Gelrite, Sigma, Milano, Italy) was added to a final concentration of 0.8%, and MgCl_2_ and CaCl_2_ were added to 3.0 and 1.0 mM, respectively. Aliquots from frozen cultures were seeded on plates and grown as circular colonized areas, as previously described [27]. Cells on plates were inoculated in a liquid medium (50 mL) in Erlenmeyer flasks and incubated in a water bath at 80 °C under orbital shaking (140 rpm), and a suitable 1 to 10 scaling up of the cultures was performed before the cells reached the stationary phase (about 1.0 OD_600_). For large-scale preparation, cells were cultivated in a 10 L fermenter (Biostat C, B. Braun Biotech International, Melsungen, Germany) with oxygen bubbling at a constant rate (0.5 L culture^−1^ min^−1^) and 140 rpm agitation.

For small-scale preparation (up to 1 L cultures) of the free extracellular proteins, equal volumes of each culture (PYS, TYS and YS) were withdrawn at late exponential phases (at about 2.0, 1.5 and 1.2 OD_600_, respectively), harvested by centrifugation at 4000× *g* for 10 min at 4 °C, and the supernatant was concentrated by ultrafiltration through Amicon membranes (cut-off: 10,000 Da) and analyzed by 12.5% polyacrylamide SDS-PAGE, according to Laemmli *et al.* [28]. All the experiments were based on at least three different protein preparations.

### 3.3. Sequence and Structural Analysis

Automated *N*-terminal degradation of the purified proteases electroblotted onto polyvinylidene difluoride (PVDF) membranes (Bio-Rad, Milano, Italy) was performed using a Perkin-Elmer Applied Biosystems 477A pulsed-liquid protein sequencer equipped with a 120A model phenylthiohydantoin analyzer for the online identification and quantification of phenylthiohydantoin amino acids

The sequence database was searched using the BLAST-PSI program [29], and the proteins identified were compared with orthologs from both Archaea and Bacteria. Multiple sequence alignments and identity scores were generated by using the MUSCLE algorithms contained in the CLC Main Workbench 6.9 program (CLC bio, 2013, Aarhus, Denmark). Protein domain organization and the prediction of transmembrane topology were obtained by using the SMART (http://smart.embl-heidelberg.de/) [30] and PSIPRED Protein Sequence Analysis Workbench (http://bioinf.cs.ucl.ac.uk/psipred/) [31] programs.

### 3.4. Purification of the SSO1175 Protein

For large-scale production, proteins from 10 L of filtered culture broth (from TYS medium only) were precipitated by the addition of (NH_4_)_2_SO_4_ up to 90% saturation at 4 °C and centrifuged at 12,000× *g* for 30 min. The protein pellet was resuspended in 50 mM Tris HCl pH 7.0 and dialyzed against the same buffer overnight at 4 °C. The protein solution was then loaded onto an anionic exchange chromatography column of DEAE-cellulose (DE52, 2 × 24 cm; Whatman, Pittsburgh, PA, USA), equilibrated with 50 mM Tris HCl buffer pH 7.0. Bound proteins were eluted by a 400 mL linear gradient of 0.0–1.0 M NaCl in the equilibration buffer, at a flow rate of 1.2 mL/min. Fractions containing the protein of interest, monitored by protease activity and SDS-PAGE, were pooled, concentrated on an Amicon PM-10 membrane (EMD Millipore, Billerica, MA, USA) and dialyzed against 50 mM Tris HCl buffer, pH 8.0, containing 1.0 M (NH_4_)_2_SO_4_ and applied to a HiTrap Phenyl Sepharose HP column (Amersham, Pittsburgh, PA, USA), pre-equilibrated with the same buffer. The column was washed at a flow rate of 1 mL/min. and the elution was performed with a linear gradient of (NH_4_)_2_SO_4_ from 1 to 0 M in 50 mM Tris HCl buffer, pH 8.0. The collected fractions containing the purified protein were pooled, desalted and concentrated for further characterization.

### 3.5. Protease Activity Assays

Protease activity was assayed using bovine hemoglobin as the substrate, monitoring the release of trichloroacetic acid-soluble peptides. The hemoglobin assay mixture contained 6.25 mg of hemoglobin in 0.5 mL of 100 mM sodium formate buffer, pH 3.0; the reaction was started by the addition of the protease sample (from 4 to 10 μg of total proteins) with incubation at 70 °C, stopped after 20 min by adding 1 mL of 5% Trichloroacetic acid (TCA) and chilling the mixture on ice. After centrifugation at 16,000× *g* for 10 min at 4 °C, the supernatant was recovered, and the peptide amount was determined by Bio-Rad protein assay. The enzyme sample was replaced with water for the blank. One unit of protease activity was defined as the amount of enzyme that produced the release of 0.1 μg of peptides, after 20 min at 70 °C and pH 3.0. Peptidase activity was assayed by using the chromogenic substrate, N-CBZ-glycine *p*-nitrophenyl ester (Z-gly pNPE) (Sigma). The assay mixture contained 0.1 mM of the substrate, in a final volume of 1 mL of 100 mM sodium formate buffer, pH 3.0. After preincubation for 10 min at 70 °C, the reaction was started by the addition of the protease samples. Hydrolysis of the substrate was monitored following the absorbance increase at 404 nm at 70 °C against a blank without enzymes.

The effects of different protease inhibitors on protease activity were tested by preincubating the enzyme sample with the different putative inhibitors, in 25 mM formate buffer, pH 3.0, for 30 min, before the addition of the substrate, as described in the protease assay section. The following protease inhibitors (all from Sigma) were used: PMSF, EDTA and pepstatin A.

### 3.6. Protease Analysis by Zymography

Detection of specific proteases on gels was performed in 8% SDS-PAGE containing 0.1% gelatin; the samples were loaded directly after the addition of the sample buffer [28], after 60 °C pre-warming (60 min) or after 5-min thermal denaturation at 100 °C. After electrophoresis, gels were washed two times with 2.5% Triton X-100 for 60 min, 3 times in distilled water for 15 min, equilibrated in 200 mM formate buffer, pH 3.0, and then incubated in the same buffer for 1 h at 70 °C. The gels, fixed in a 50% TCA solution for 10 min at 25 °C, were then stained with Coomassie brilliant blue G-250 for 20 min. After destaining, clear bands on the blue background represented the gelatinolysis areas.

Alternatively, the detection of specific proteases on gels was performed in 8% native PAGE containing 0.1% gelatin. In this case, after electrophoresis, gels were washed 3 times in distilled water for 15 min, equilibrated in 200 mM formate buffer, pH 3.0, and then incubated in the same buffer for 1 h at 70 °C. The staining was performed as described above. Concentrated samples of PYS and TYS broth cultures were loaded as controls.

### 3.7. Substrate Specificity Assay on the Oxidized Insulin B Chain

The oxidized B chain from bovine insulin (Sigma) was used as substrate for the purified protease from TYS cultures. Reactions were performed according to Palmieri *et al.* [23] by incubating 0.1 μg of protease in the presence of the insulin B chain (0.05 mg) at 70 °C in 0.12 mL of 5% formic acid, pH 3.0, for 30 min. Aliquots of 60 μL were removed from the reaction mixtures at 0 and 30 min of incubation time and mixed with 5 μL of 5% TFA to stop the reaction. Proteolytic mixtures were analyzed each time by liquid chromatography-mass spectrometry-mass spectrometry (LC-MS-MS) analysis using an ion trap mass spectrometer (Agilent Technologies, Santa Clara, CA, USA) coupled with nano-HPLC-chip systems, using a microfluidic chip-based technology specifically designed for nanospray LC/MS. After loading, the peptide mixture (3 μL) was first washed and concentrated onto a reverse-phase pre-column present on the chip using 0.2% formic acid and 2% acetonitrile as the eluent at 4 μL/min. The sample was then fractionated onto the same chip by a C18 reverse-phase capillary column at a flow rate of 300 nL/min using a linear gradient of eluent B (0.2% formic acid in 95% acetonitrile) in A (0.2% formic acid in 5% acetonitrile) from 7% to 60% in 50 min. The mass spectrometer was set up in a data-dependent MS/MS mode, where a full scan spectrum (*m*/*z* acquisition range from 400 to 1600 Da/e) was followed by a tandem mass spectrum in the *m*/*z* acquisition range, depending on the selected parent ion.

### 3.8. Phylogenetic Analysis

The amino acid sequences of SSO1175 and SSO2045 were each compared with the NCBI non-redundant protein dataset (the version available on 28 October 2013) by running the Blast 2.2.18 program available at http://www.phylogeny.fr/ [29,32], in order to identify public, similar sequences. The program was run with the default parameters. For each run, proteins corresponding to hits with a BLAST *e*-value lower than 1 × 10^−20^ and covering at least 50% of query sequence were selected. A MUSCLE alignment [33] of the selected sequences was analyzed by the ProtTest 3.3 program [34] to find the best-fitting protein evolution model. The model and parameters identified by ProtTest 3.3 were utilized for the “One Click Mode Phylogeny analysis” pipeline, available on Phylogeny.fr, which runs the following programs on the input sequences: MUSCLE for multiple alignment, Gblocks for alignment curation [35] and PhyML for phylogeny [36]. At the end of the procedure, a unique phylogenetic tree was obtained, which was edited by the PhyloWidget web program [37].

## 4. Conclusions

In this work, the extracellular proteolytic activities of *S. solfataricus* during its growth in complex peptide media were analyzed. Our aim was to identify new extracellular proteases from cultures grown in the presence of peptone or tryptone when added to a yeast extract/sucrose-based medium. In these culture conditions, we found an increase in the cell growth rate and in the production of peptidolytic systems. Therefore, specific regulation mechanisms could be set up by *S. solfataricus* to finely regulate and coordinate the expression of genes encoding different components of the proteolytic system. In this context, two cell-associated thermopsin-like proteases, SsMTP-1 and the previously identified SsMTP, showing unusual domain organization and different cleavage site specificities, were found to be overproduced in peptide-rich media, thus suggesting their involvement in the degradation of proteinaceous substrates. Moreover, these proteases clustered into different thermopsin phylogenetic groups within the *Crenarchaeota* phylum and were predicted to be implicated in cell signaling transduction processes, indicating their possible pivotal role in driving microbial functional diversity and adaptative environmental functioning in extreme living ecosystem.

## Supplementary Information
