# Supplementary material for: A New Pepstatin-Insensitive Thermopsin-Like Protease Overproduced in Peptide-Rich Cultures of Sulfolobus solfataricus
Source: Int J Mol Sci. 2014 Feb 21;15(2):3204–19. doi: 10.3390/ijms15023204 (PMC3958906; doi:10.3390/ijms15023204)
Supplement: Supplementary file 1 [file ijms-15-03204-s001.pdf]

Supplementary Information

Table S1. Summary of purification of 75 kDa-gelatinase from tryptone, yeast extract and sucrose (TYS) culture medium (10 L) of *Sulfolobus solfataricus* P2

Purification step	Volume (mL)	Protein (mg)	Activity (U)	Yield (%)	Specific activity (U/mg)	Purification (fold)
$(NH_4)_2SO_4$	50	21.0	13,000	100	619	1.0
DEAE-cellulose	5	3.0	7,410	57	2,470	4.0
Phenyl sepharose	1	0.2	1,400	11	7,000	11.3

Table S2. Peptides with the corresponding mass values, released from the oxidised insulin B chain following the proteolysis experiment with *Sulfolobus solfataricus* multi-domain thermopsin-like protease-1 (SsMTP-1). The fragments 1-30 represents the undigested oxidised insulin B chain.

Peak	Peptide	Measured mass value	Expected mass value
1	8–21	1538.8	1538.7
2	3–7; 24–28	662.7; 673.7	662.7; 673.8
3	24–30	873.1	873.0
4	16–30	1798.8	1798.8
5	1–30	3494.1	3494.0

Figure S1. Muscle alignment of *Sulfolobus solfataricus* multi-domain thermopsin-like protease (SsMTP) and SsMTP-1 sequences with the homologues retrieved in the available archaeal organisms database. The consensus sequence, the conservation histogram and the sequence logo are shown at the bottom of the alignment.

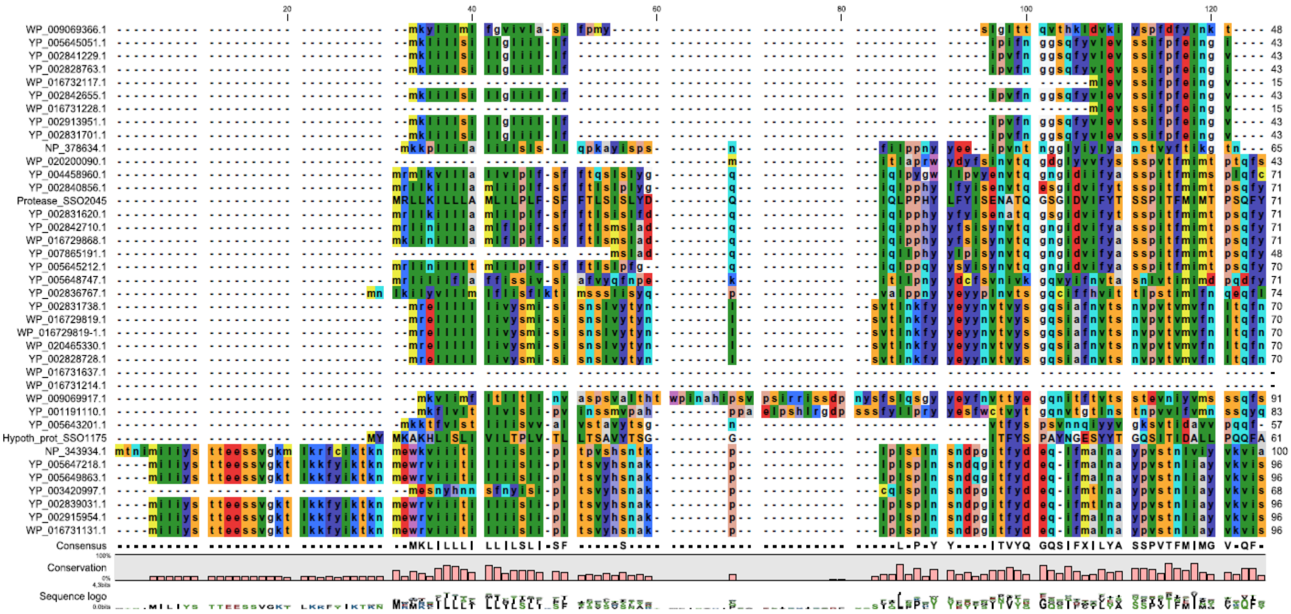

Figure S1. *Cont.*

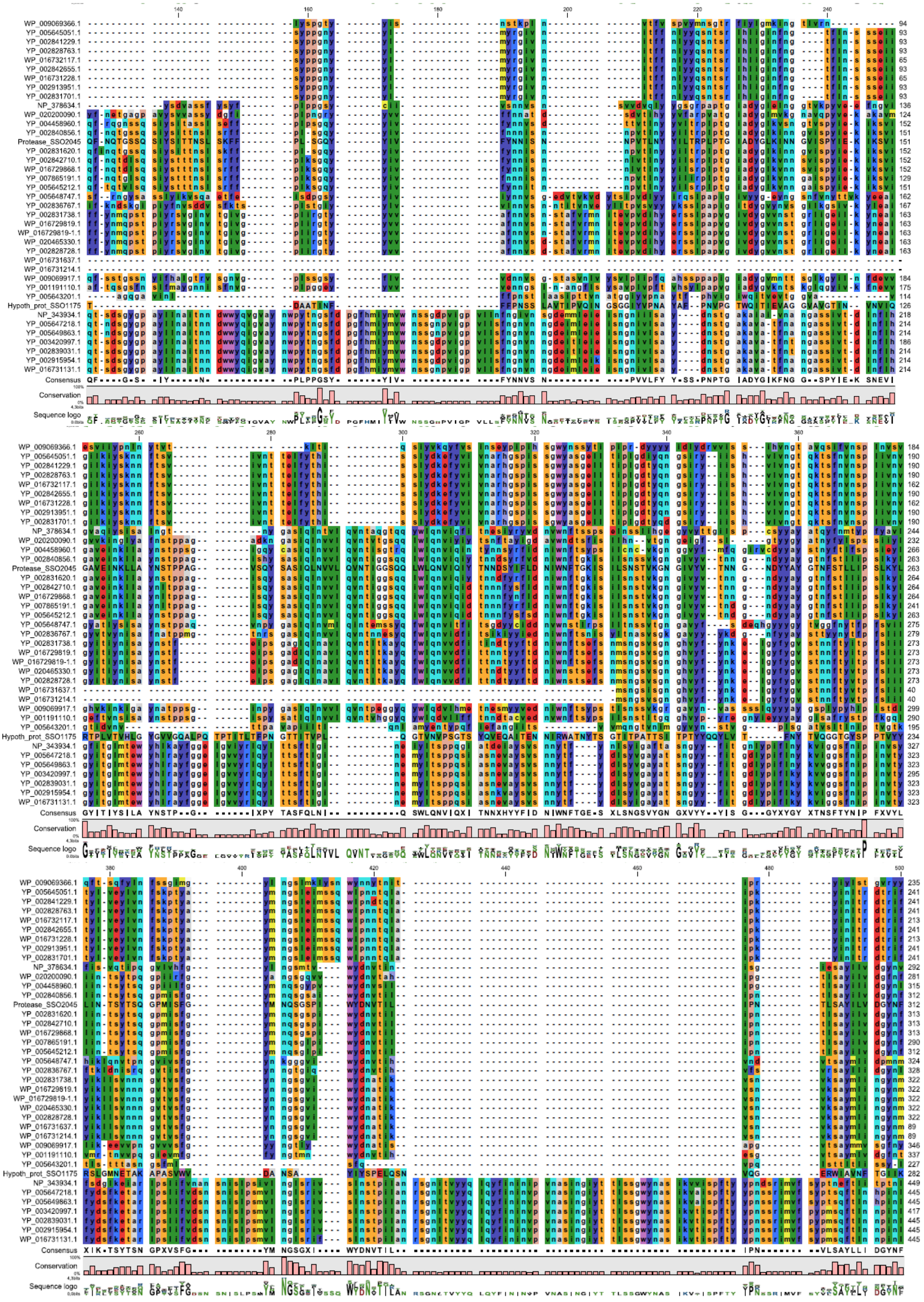

Figure S1. *Cont.*

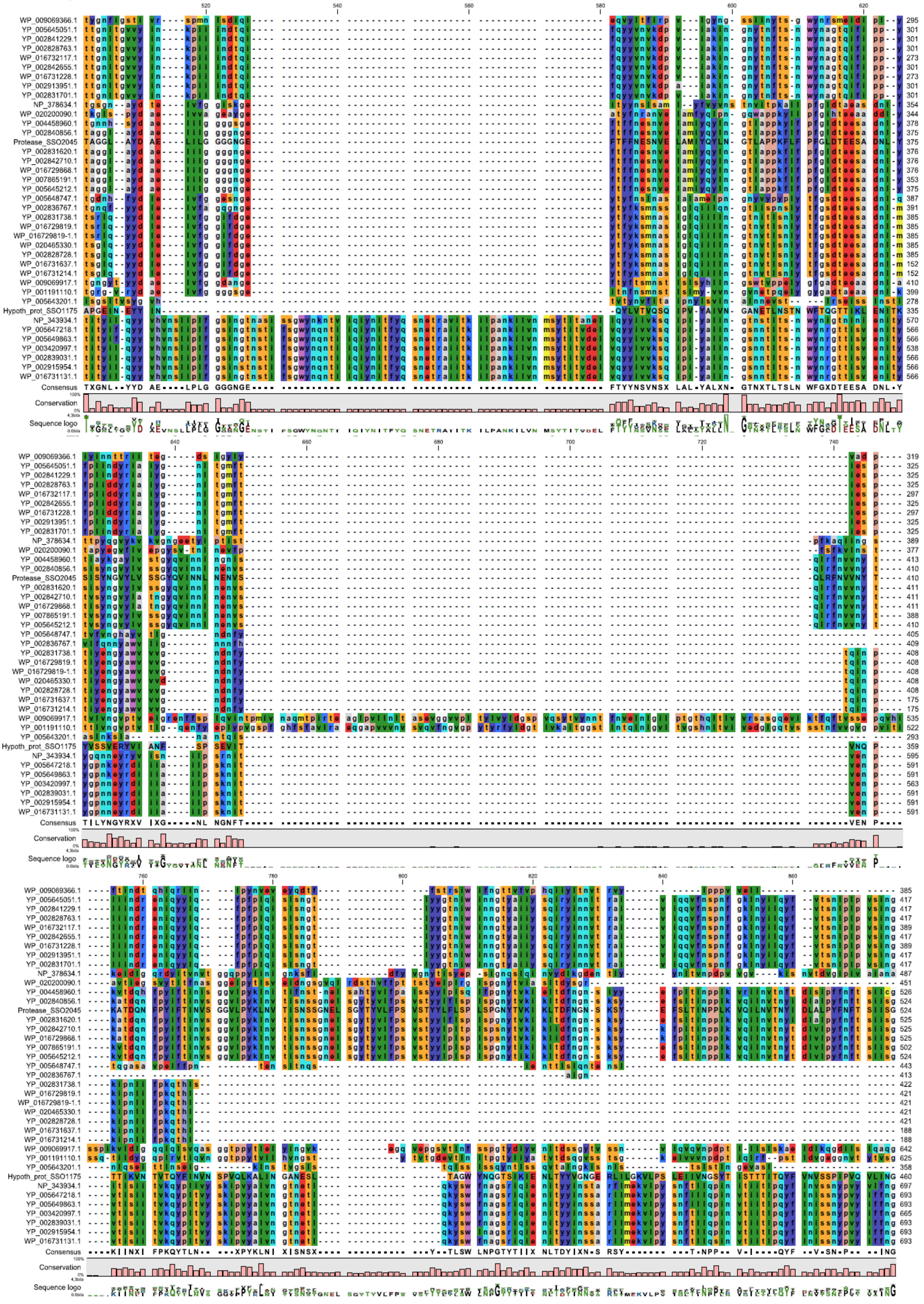

Figure S1. *Cont.*

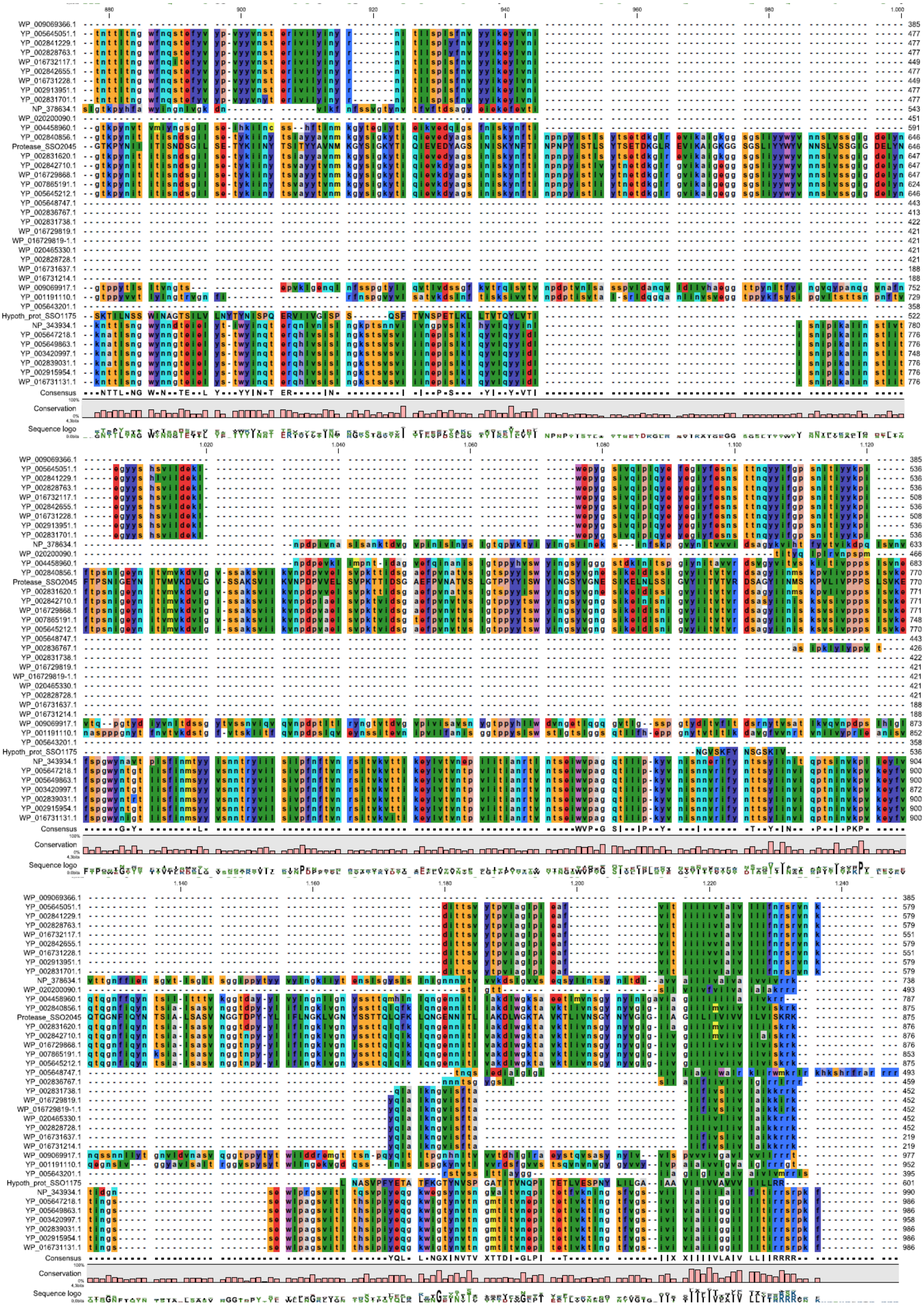

Figure S2. Molecular properties of SsMTP-1. (**A**) The influence of temperature on enzyme activity; (**B**) The pH-activity profile; and (**C**) The thermoresistance of SsMTP-1 at different temperatures. All experiments were performed in triplicate on three different protein preparations, using N-CBZ-glycine *p*-nitrophenyl ester as substrate. SD values lower than 5% are not shown.

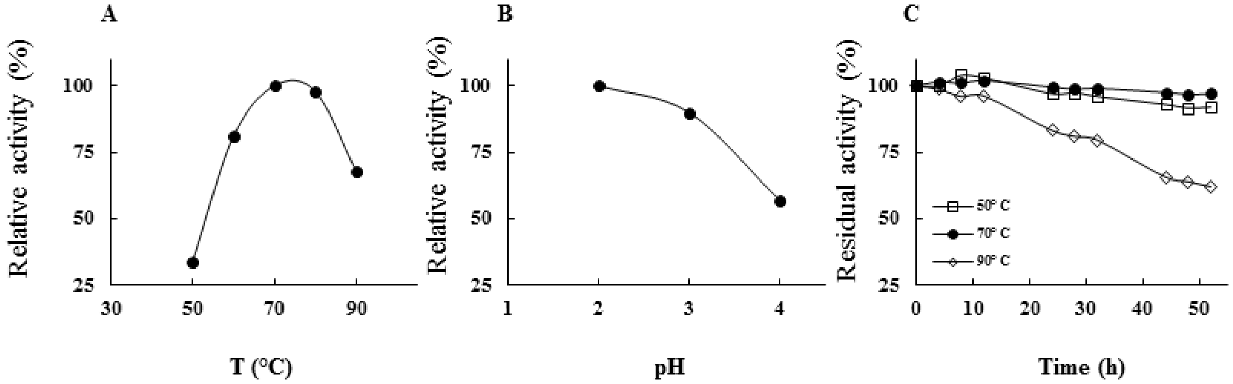

© 2014 by the authors; licensee MDPI, Basel, Switzerland. This article is an open access article distributed under the terms and conditions of the Creative Commons Attribution license (http://creativecommons.org/licenses/by/3.0/).
